# Traumatic Right Coronary Artery Dissection as a Cause of Inferior Wall ST-Elevation Myocardial Infarction

**DOI:** 10.7759/cureus.6694

**Published:** 2020-01-18

**Authors:** Vrinda Vyas, Madhuri Badrinath, Tamas Szombathy

**Affiliations:** 1 Internal Medicine, State University of New York (SUNY) Upstate Medical University, Syracuse, USA; 2 Cardiology, State University of New York (SUNY) Upstate Medical University, Syracuse, USA

**Keywords:** traumatic corornary artery dissection, coronary dissection, blunt chest trauma, coronary angiography, coronary intervention, coronary artery

## Abstract

Blunt cardiac injury, causing coronary artery dissection in the absence of other forms of injury to the heart or lungs is a rare occurrence. Here we present a case of a 41-year-old male who presented with right coronary artery (RCA) dissection after blunt chest trauma. The patient initially presented with chest pain and was diagnosed with an inferior wall myocardial infarction (MI). He then developed a complete heart block and bedside echocardiogram showed right ventricular akinesis. Immediate coronary angiography showed RCA dissection, and TIMI 3 flow was established after the placement of four drug-eluting stents. Blunt trauma-induced RCA dissection is associated with high mortality which needs immediate treatment. Hence through this case report, we would like to stress the importance of having a high index of suspicion for this condition in patients with a blunt chest injury.

## Introduction

Common causes of an inferior wall myocardial infarction (MI) include atherosclerotic disease secondary to hypertension and diabetes, coronary artery spasms and inflammatory systemic diseases. However, traumatic coronary artery dissection from blunt thoracic trauma, leading to an acute MI has a low incidence [1,2]. In patients who sustain a chest trauma and develop persistent chest pain without any signs of hemopericardium, haemothorax or pneumothorax, it is crucial to have a high index of suspicion for coronary artery trauma. The diagnosis may be especially difficult in the absence of an electrocardiogram (EKG) because chest pain following a blunt thoracic injury can be interpreted as being secondary to chest wall contusion, rib fractures, or it may be overlooked in the presence of other injuries. EKG changes such as hyperacute T-waves in the distribution of the affected territory, which might evolve into ST-segment elevation, are suggestive of a coronary pathology [1]. Hence, EKG should be performed in all patients with thoracic trauma. Amongst the coronary arteries, right coronary artery (RCA) is less frequently injured in blunt cardiac trauma, with left anterior descending (LAD) artery being the most injured [1,2]. We present a case of a 41-year-old male who presented with a traumatic RCA dissection leading to inferior wall ST-elevation myocardial infarction (STEMI). The management of traumatic coronary artery dissection remains controversial and there is no established standard of care [1].

## Case presentation

**Figure 1:**
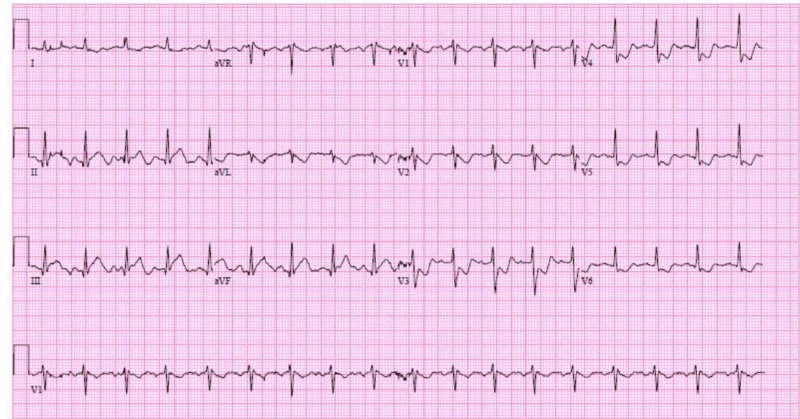
Electrocardiogram (EKG) on presentation shows ST elevations in leads II, III and aVF

A healthy 41-year-old male with no past medical history apart from alcoholism and smoking presented with chest pain after a high-speed motor vehicle accident and steering wheel trauma to the chest. On admission, his vitals were stable and physical examination was unremarkable. EKG showed ST elevations in leads II, III and aVF consistent with inferior wall MI and troponin was elevated at 5.38 ng/ml (Figure 1).

**Figure 2:**
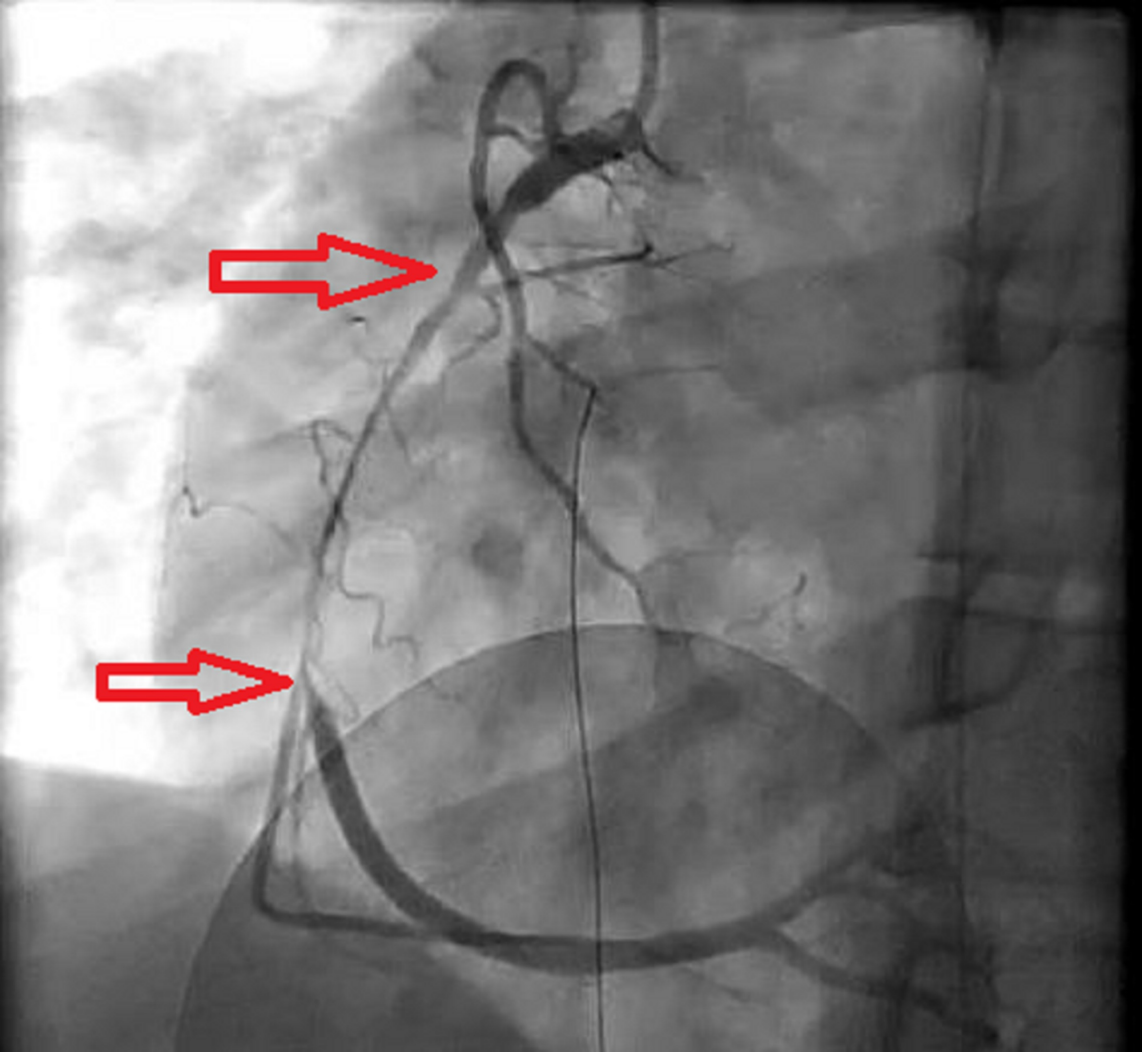
Coronary angiography showing a lesion in the right coronary artery (RCA) with 99% stenosis (TIMI 1 flow)

Before any other diagnostic tests could be performed, the patient developed severe substernal chest pain and soon became confused. EKG showed a complete heart block causing hemodynamic instability that required immediate transcutaneous pacing. An urgent bedside echocardiogram showed right ventricular akinesia with no evidence of pericardial effusion or other structural injuries to the heart. The patient was urgently taken to the cardiac catheterization laboratory. Coronary angiography revealed a long intimal flap in the RCA resulting in a 99% stenosis of the vessel, without resolution on intracoronary nitroglycerin administration (Figure 2). The remaining coronaries were normal.

**Figure 3:**
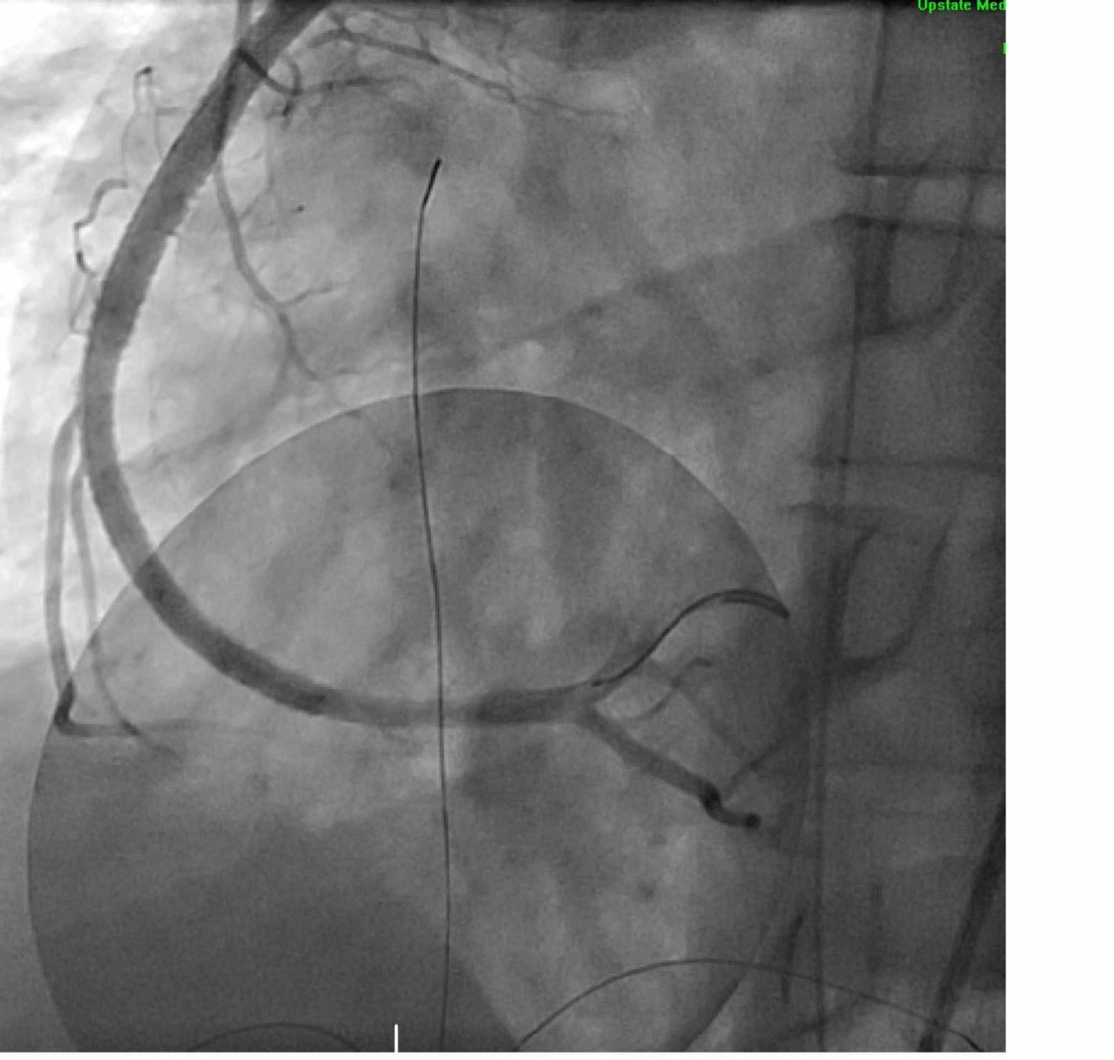
Coronary angiography image after placement of four drug eluting stents into the right coronary artery (RCA). RCA now has 0% stenosis (TIMI 3 flow)

He underwent percutaneous coronary intervention, with the subsequent deployment of four drug-eluting stents with improvement from initial 99% stenosis (TIMI 1flow) to final 0% stenosis with TIMI 3 flow (Figure 3). Further imaging showed no evidence of other forms of blunt trauma injury like a cardiac contusion, hemopericardium, hemothorax or pneumothorax. The patient was monitored in the cardiac ICU and once medically stable, was discharged on dual antiplatelet therapy and a beta-blocker.

## Discussion

Clinically significant cardiac manifestations of blunt chest injury occur in 5-15% of patients and range widely from clinically silent, conduction system abnormalities, myocardial contusion, to cardiac wall rupture. Isolated coronary artery dissection following blunt chest injury in the absence of other forms of cardiac injury is an uncommon and life-threatening complication [1]. To date, there have been very few reported cases of RCA dissection with an incidence of 12% compared to left anterior descending artery dissection, which is more commonly affected due to the proximity to the chest wall with an incidence of 76% [2].

The mechanisms of dissection include intimal tear with propagation to the media and the torn intima then creates a flap that obstructs the blood flow and produces infarction; intramural thrombosis and coronary vasospasm may also play a role [3]. The origin of the right coronary artery may be particularly vulnerable to blunt trauma because the proximal right coronary artery moves anteriorly towards the sternum during systole [4,5]. However, there is no clear relationship between the severity of chest wall trauma and the development of coronary lesions, making these difficult to suspect [5].

Coronary artery dissections have a wide variety of presentations; from being asymptomatic to resulting in acute coronary syndrome and sudden cardiac death [5]. The diagnosis of traumatic coronary artery injury may be missed as it is rare and the time difference from the actual chest injury to coronary artery trauma can vary from 48 hours sometimes up to five weeks [6]. Hence, the initial diagnostic evaluation of polytrauma patients with a chest injury and/or chest pain should include ECG, serial cardiac enzymes and continuous cardiac monitoring [3,5]. Patients with chest trauma, have elevated troponin levels, which could be either from structural cardiac injury (such as myocardial contusion, myocardial rupture) or from skeletal trauma, and it is important to distinguish these from elevation in cardiac troponin due to myocardial infarction from coronary artery injury. An elevated level of cardiac troponin with concomitant hyperacute T waves and/or ST-segment elevation in a patient with blunt chest trauma most likely represents coronary vessel injury and should lead the physician to investigate it further. An echocardiogram can be an instrumental tool to differentiate between structural and non-structural damage to the heart as a result of chest trauma [3]. It might also demonstrate regional wall motion abnormalities when there is coronary artery occlusion, just as it did in our patient, whose Echo revealed complete akinesis of RV further corroborating the diagnosis of traumatic acute myocardial infarction.

Coronary angiography is the diagnostic tool of choice to prove coronary dissection and simultaneously allow for successful treatment [6]. Other methods of diagnosis which have been described include multi-detector row computed tomography, cardiac magnetic resonance imaging, and intravascular ultrasound [7-9]. Traumatic coronary artery dissection can be managed with coronary artery bypass grafting, balloon angioplasty, angiography with stent placement, conservative medical management, or thrombolysis based on careful patient selection. Success rates for these treatment modalities are difficult to ascertain due to the small sample size and limited patient follow-up [10].

## Conclusions

Our patient presented to the hospital with classic acute coronary syndrome triad (chest pain, ST elevation in inferior leads and elevated troponin level) following trauma to the chest and had to be emergently taken for coronary angiography after he quickly decompensated as a result of new complete heart block from the acute inferior wall STEMI. Because of timely intervention, our patient had only minor impairment of cardiac function at discharge from the cardiac intensive care unit. There have been case reports of missed or delayed diagnosis. Hence, we aim to highlight that although the recognition of traumatic coronary artery dissection can be difficult because of varied clinical presentation and mechanisms of trauma, delayed presentation to the hospital and lack of guidelines in assisting with the diagnosis and treatment, it should always be considered in patients with blunt thoracic trauma who have chest pain on presentation.
